# “Loneliness is killing me?!”: the subjective emotional experience of loneliness during the COVID-19 pandemic: results of a cross-sectional study in patients with a psychiatric disorder

**DOI:** 10.1007/s00127-024-02808-w

**Published:** 2024-12-23

**Authors:** Mona Schenk, Sabrina Baldofski, Fabian Hall, Tony Urbansky, Maria Strauß, Elisabeth Kohls, Christine Rummel-Kluge

**Affiliations:** 1 Department of Psychiatry and Psychotherapy, Medical Faculty, Leipzig University, Semmelweisstr. 10, Haus 13, Leipzig, 04103 Germany; 2 Department of Psychiatry and Psychotherapy, University Leipzig Medical Center, Leipzig, Germany

**Keywords:** Loneliness, Mental disorder, COVID-19, Psychiatric patients, Life satisfaction, Interpersonal trust

## Abstract

**Purpose:**

During the COVID-19 pandemic, an increase in loneliness as well as mental health issues was detected. However, research on the association between loneliness and mental disorders is sparse. The aim of this study was to examine loneliness and associated social and emotional factors in patients with a psychiatric disorder and to investigate potential predictors of loneliness.

**Methods:**

Participants were *N* = 230 patients currently receiving psychiatric treatment at the Department of Psychiatry and Psychotherapy, University Hospital Leipzig, Germany. A cross-sectional survey included questionnaires on loneliness, life satisfaction, need to belong, interpersonal trust, stress, and resilience.

**Results:**

Most participants (*n* = 91, 39.6%) suffered from depression, followed by anxiety disorder (*n* = 43, 18.7%). Significantly higher loneliness levels compared to norm samples were detected in all three loneliness questionnaires (all *p* <.05), and overall *n* = 128 (57.7%) reported to feel lonely. In addition, participants reported lower life satisfaction, lower interpersonal trust, and lower resilience than the general population (all *p* <.05). No significant differences in loneliness levels between different psychiatric diagnoses were revealed. It was found that lower satisfaction with life, lower interpersonal trust and lower resilience were significantly associated with higher loneliness (all *p* <.05).

**Conclusion:**

This study underlines the importance to continue research on loneliness in people with mental disorders after the COVID-19 pandemic since the majority of patients reported to feel lonely. Further, tailored therapy-accompanying interventions to prevent loneliness in patients with a psychiatric disorder should be designed and evaluated to meet patients’ diverse needs e.g., through online programs.

**Trial registration:**

German Clinical Trial Registration: DRKS00023741 (registered on April 6, 2021).

## Introduction

When COVID-19 started to spread worldwide in 2020, the extraordinary circumstances of lockdowns, social distancing measures, and closings of many public institutions had consequences on people’s personal lives. In particular, loneliness is becoming a growing public health issue in today’s society [1, 2]. Defined as a “loneliness epidemic”, the rising social detachment is associated with many negative physical and emotional effects [3, 4].

Loneliness describes a subjective, negative emotional experience which occurs due to an imbalance between the need for social interaction and the actual quantity and/or quality (e.g. meaningfulness, closeness) of social relationships [5, 6]. Several studies showed that a two-dimensional approach on loneliness, classifying it into emotional and social loneliness, is important given the different psychological and emotional challenges related to these two domains [7, 8]. Emotional loneliness refers to a lack of meaningful relationships and someone to turn to, whereas social loneliness describes an absence of a social network such as a group of acquaintances, resulting in an absent sense of belongingness [9].

Since the beginning of the COVID-19 pandemic, an increase in loneliness and social disconnection has been detected [10] as well as a rising trend in alcohol and drug abuse, anxiety, depression, suicidal ideation, anger, and stress [11–13]. Suicide rates were not as elevated as expected [14, 15]. However, in contrast to decreasing suicide rates in the past years, recent research shows an upward trend in Germany, especially in people aged 60 and above [16–19]. Particularly adults living alone, people with low income, and elderly persons (often living alone and engaging in less social interaction [3]), who were already at risk for being affected by loneliness before the pandemic, were even more severely impacted by social restriction rules [20, 21]. A longitudinal Spanish study found that young age and a pre-pandemic existing mental disorder were the main risk factors to suffer from loneliness [22]. Further, college or university students had a heightened risk for loneliness during the pandemic in the UK [20], as well as in Switzerland and Germany where studies additionally showed an increase in anxiety and depressive tendencies [23–25]. Australian adolescents reported higher levels of both depressive symptoms and feelings of loneliness after COVID-19 school closings in a longitudinal study [26]. However, there are to date only few studies investigating epidemiological factors regarding loneliness during the pandemic in patients with a psychiatric disorder. These studies suggest that being single and a psychiatric diagnosis itself heightened the risk for loneliness during pandemic-related social restrictions [27]. Further, the results support the bidirectional relationship between loneliness and mental health as loneliness was found to be a risk factor for depression and anxiety [28, 29].

Aside from epidemiological factors associated with loneliness (e.g. age < 30 years and > 65 years [3, 30], female gender, low income) [20], it has also been found that people experiencing loneliness are more likely to experience physical health risks [31] and were reducing their physical activity [32] in longitudinal studies. A study from the United States indicated that social isolation has a bigger effect on hypertension than clinical risk factors (e.g. diabetes) [33], while simultaneously increasing cardiovascular risk factors (e.g. Body-Mass-Index, high blood pressure) [34]. Concerning the risk for early death, the predictive effects of a lack of social support and social engagement are comparable to those of established mortality risk factors such as smoking or physical inactivity [35, 36].

Although previous research showed that loneliness plays an important role in the development of and maintaining mental disorders [37, 38], there are only few studies further focussing on this association [39]. Two cross-sectional studies in patients with a psychiatric disorder confirmed this connection [40, 41], showing that especially patients with depression felt lonelier and suffered more from loneliness than people without psychiatric disorders. In general, several cross-sectional studies discovered an association between feeling lonely and many mental disorders, such as personality disorders, psychosis, depression, anxiety [34, 42–44], and suicidal ideation [45]. It has been reported that loneliness is not only predicting mental disorder in cross-sectional and longitudinal studies [46], but a meta-analysis found that mental distress itself leads to higher loneliness [47]. A Dutch longitudinal study confirmed this bidirectional relationship and found that loneliness predicts both the onset and the persistence of mental disorders [38]. In this context, it is important to mention that stigma and discrimination associated with mental health can lead to people feeling isolated, which in turn may cause feelings of hopelessness, leading to suicidal ideation, and suicide attempts [41, 48]. A recent study discovered that patients with schizophrenia suffered severely from internalised stigma resulting in social withdrawal and a decreased self-esteem, eventually leading to avoidance of psychiatric support and a worsening prognosis [49].

There are several social and emotional factors that can be linked to loneliness. According to previous cross-sectional studies, interpersonal trust and life satisfaction are negatively associated with loneliness [50, 51]. Furthermore, the relationship between life satisfaction and a person’s unmet need to belong was found to be mediated by loneliness, suggesting that people with a higher need to belong tend to feel less satisfied with life [52] because they are lonely. Further, stress and resilience are other important factors associated with loneliness. Stress, as well as loneliness, are known to play an important role in the aetiology of physical health problems, such as glucocorticoid resistance or oxidative stress [53]. Moreover, an analysis showed that people feeling more lonely are also more likely to experience stress in everyday events compared to socially connected individuals [54]. Studies also indicated that social support could reduce stress levels [55, 56]. Although there are only a few studies on the connection between loneliness and resilience (defined as the ability to withstand or recover quickly from experiencing distress [57]), findings of cross-sectional studies demonstrate that a higher resilience was associated with lower loneliness levels, decreased anxiety, and an increased quality of life [58–60].

Observing the rising recognition of loneliness as a public health problem [1] and given the rather small number of existing studies on loneliness and its association with mental disorders [39], the aim of this cross-sectional study was to comprehensively analyse loneliness levels in patients with a psychiatric disorder during the pandemic compared to the general population, as well as participants’ life satisfaction, interpersonal trust, need to belong, stress, and resilience. Further, the association between loneliness and different psychiatric diagnoses was examined, and potential predictors of loneliness were analysed. It was hypothesized that (1) patients with a psychiatric disorder would be more likely to report experiencing higher levels of loneliness than the general population, along with higher need to belong and stress, and lower life satisfaction, interpersonal trust and resilience, and that (2) gender, age, living situation, occupation, diagnosis, life satisfaction, need to belong, interpersonal trust, perceived stress and resilience are expected to be significant predictors of loneliness.

## Materials and methods

### Participants and procedure

This prospective study was conducted between June 2021 and June 2022 at the Department of Psychiatry and Psychotherapy of the University Hospital Leipzig, Germany. During this period, current pandemic-related governmental restrictions in Germany included only being allowed to enter the work place, events or the psychiatric outpatient clinic when either being vaccinated, being negatively tested for, or being recovered from COVID-19. Further, protective measures like wearing a face mask, quarantine for contact persons and a recommendation to work from home applied.

This study’s participants were recruited from inpatient wards, the psychiatric outpatient clinic, and the psychiatric day clinic in the University Hospital Leipzig. To be included in the study, participants had to be 18 years or older, to be able to complete the survey without help and to currently receive treatment in the psychiatric department. Patients were excluded in case of illiteracy or visual impairment, inadequate German language skills, or an acute suicidal crisis which was determined by the treating psychiatrist.

The study was first presented to inpatients and day hospital patients within their therapies. Outpatients were first introduced to the study in the outpatient clinic’s waiting area. Patients were approached and verbally informed about the study, including information about data protection and the approximate length of the survey. In case a patient was interested in study participation, inclusion and exclusion criteria were checked and the patient was given a handout and a consent form. They were reapproached after some time for consideration. If patients met the inclusion criteria, they gave written informed consent before participating in the study.

Participants were presented with two options for completing the survey, either the paper-and-pencil or the online version of the questionnaire. If they chose the pen-and-paper questionnaire, they were handed the questionnaire and were given approximately 40 min for completion, or longer if needed. If they chose the online questionnaire, they received an information sheet including a hyperlink leading to the survey (survey software by Unipark) which they could then complete at any time according to their preferences. *N* = 520 patients were approached, of which *n* = 411 (79.0%) gave written informed consent to participate in the study. In total, *n* = 230 (44.2%) patients participated in the survey, of which *n* = 209 (90.9%) completed the survey and *n* = 21 (9.1%) quit the survey at different stages. Overall, *n* = 67 (29.1%) of those who participated chose the paper-and-pencil version and *n* = 163 (70.9%) chose the online version.

The Ethical Committee of the Medical Faculty of Leipzig University approved this study on October 6, 2020 (393/20-ek). The study was registered at the German Clinical Trials Register (DRKS00023741).

### Measures

#### Sociodemographic information

In the first section of the questionnaire, sociodemographic data (gender, age, marital status, living situation, educational level, current occupation) were assessed.

#### Clinical information

Additionally, the clinician-rated diagnosis for which treatment was currently being received was used. It has been assigned initially during a standardized diagnostic procedure by experienced psychiatrists (in training) and licensed clinical psychologists (including clinical interviews and standardized self-report measures). Participants were allocated to one of six groups based on their main psychiatric diagnosis. These six groups were (according to ICD-10): schizophrenia (F20-F29), bipolar disorder (F31), unipolar depression (F32, F33), anxiety and obsessive-compulsive disorder (F40-42), personality disorder (F60-F69), and attention deficit disorder/ attention deficit hyperactivity disorder (F90-F98).

#### Loneliness

The second section of the questionnaire evaluated the subjective emotional experience of loneliness. In total, three questionnaires were included since the study aimed to assess the construct of loneliness thoroughly, including different aspects of loneliness.

Using the German version of the revised UCLA Loneliness Scale (“Hamburger Einsamkeitsskala”) [61], the participants answered 20 items on Likert scales ranging from 1 = “I never feel this way” to 4 = “I often feel this way”. Higher sum scores indicated higher levels of loneliness. A commonly used categorisation was used: a score of 20–34 indicated a low degree of loneliness, 35–49 a moderate degree of loneliness, 50–64 a moderately high degree of loneliness, and 65–80 a high degree of loneliness [62]. The scale shows very good internal consistency (Cronbach’s α = 0.92). The norm sample data used in the following analysis can be found in the questionnaire’s original publication and represents two healthy university samples [61].

Next, the UCLA 3-item Loneliness Scale was administered [63]. This scale is not simply a short version of the UCLA Loneliness Scale, but also differs in the answering format. The participants responded to three questions on 4-point Likert scales from 0 = “never” to 3 = “often” [63, 64]. A sum score was calculated, with higher scores indicating more loneliness. A commonly used categorisation was used: a score of 3–5 indicated “not lonely” and 6–9 “lonely” [65]. The scale’s internal consistency is acceptable (Cronbach’s α = 0.79). The norm sample data used in this paper was published in the questionnaire’s original source and represented the American general population [66].

Finally, the De Jong Gierveld Loneliness Scale [67] contained a social subscale (5 items) and an emotional subscale (6 items) with 5-point Likert scales ranging from 0 = “no!” to 4 = “yes!”. Answers were dichotomised and sum scores were calculated, ranging from 0 to 11. According to the official manual, a score of 0–2 is interpreted as “not lonely”, 3–8 “moderate lonely”, 9–10 “severe lonely”, and 11 “very severe lonely” [67]. The scale evidences good internal consistency (Cronbach’s α = 0.87). Norm sample data that was used in this analysis was collected among the Dutch population and included only women [68].

#### Satisfaction with life

In a total of 5 items, the Satisfaction with Life Scale [69] (SWLS) assessed the participants’ life satisfaction on 7-point Likert scales from 1 = “strongly disagree” to 7 = “strongly agree” [69]. Higher sum scores indicated higher life satisfaction. The following categorisation was used: a score of 5–9 represented “extremely dissatisfied”, 10–14 “dissatisfied”, 15–19 “slightly dissatisfied”, 20–24 “slightly satisfied”, 25–29 “satisfied”, and 30–35 “highly satisfied” [70]. The SWLS demonstrates good internal consistency (Cronbach’s α = 0.89). The norm sample used for comparison can be found in the questionnaire’s original publication and consists of American university students [69].

#### Need to belong

The 10-item German Need to Belong Scale (NTBS) was used to evaluate the need to belong [71]. Participants gave their opinion on 4-point Likert scales ranging from 1 = “strongly disagree” to 4 = “strongly agree”. Higher scores indicated a higher need to belong [71]. The scale’s internal consistency is acceptable (Cronbach’s α = 0.77). Norm sample data and score categorisation was not available in the original publication and other sources.

#### Interpersonal trust

To measure interpersonal trust, the Interpersonal Trust Short Scale [72] (KUSIV3) was used in its original German version “Kurzskala Interpersonales Vertrauen”. It contained 3 items which were rated on 5-point Likert scales ranging from 1 = “do not agree at all” to 5 = “completely agree” and higher sum scores indicated more interpersonal trust. The scale shows good internal consistency (Cronbach’s α = 0.81). The norm sample that was used in our analysis can be found in the official manual [51, 72].

#### Perceived stress

The Stress and Coping Inventory (SCI) assesses the current stress load, stress symptoms and different coping strategies by using 54 items in ten subscales [73]. In our survey, only 53 items were used (one item in Subscale 7 was omitted by mistake). According to the authors’ recommendation, the missing value was replaced by calculating the rounded mean of the other items in this subscale. Subscale 1–3 evaluated stress due to uncertainty (7 items), stress due to overload (7 items), and stress due to loss and actual negative events (7 items). They were rated on 7-point Likert scales ranging from 1 = “not stressed” to 7 = “heavily stressed”. To determine total stress, the first three subscales were aggregated into Subscale 4. In subscale 5 (13 items), physical and psychological stress symptoms were measured on 4-point Likert scales from 1 = “I disagree” to 4 = “I agree”. In subscale 6–10 (4 items each), patients reported on coping strategies such as positive thinking, active stress coping, social support, keeping faith and increased alcohol or cigarette consumption on 4-point scales identical to subscale 5 [73]. Higher sum scores indicated higher levels of stress. The ten subscales show satisfactory internal consistency (Cronbach’s α ranging from 0.69 to 0.88). Norm sample data that was used for comparison in this analysis can be found in the questionnaire’s official manual [73].

#### Resilience

The Brief Resilience Scale (BRS) was used to assess the perceived ability to recover from stress on 5-point Likert scales ranging from 1 = “strongly disagree” to 5 = “strongly agree” in a total of six items [74]. A mean score was calculated, with higher values indicating higher levels of resilience. A commonly used categorisation was used: a score of 1–2.99 indicated low resilience, 3–4.3 normal resilience, and 4.31–5 high resilience [75]. The BRS evidences good internal consistency (Cronbach’s α = 0.84). Norm sample data used in this analysis represents university students and can be found in the questionnaire’s original publication [74].

### Statistical analysis

Statistical analyses were conducted using IBM SPSS statistics version 29.0. A two-tailed α = 0.05 was applied to all statistical tests. Descriptive statistics were used to report sociodemographic data. To evaluate the participants’ scores on different scales, official norm sample scores were used (UCLA Loneliness Scale, UCLA 3-items Loneliness Scale, De Jong Gierveld Loneliness Scale, SWLS, KUSIV3, SCI, BRS), where available cut off scores were applied in addition (UCLA Loneliness Scale, UCLA 3-item Loneliness Scale, De Jong Gierveld Loneliness Scale, SWLS, BRS). Independent *t*-tests were performed to compare the study sample’s scores with norm samples for the UCLA Loneliness Scale, UCLA 3-items Loneliness Scale, De Jong Gierveld Loneliness Scale, SWLS, KUSIV3, SCI, and BRS. Due to participants not completing the questionnaires until the end, sample sizes varied for different scales as shown in Table 1.

**Table 1:** Sociodemographic characteristics of the total sample (*n* = 230)

Variable	Total sample (*n* = 230)
**Gender**, *n* (%)	
Female	132 (57.4)
Male	91 (39.6)
Diverse	7 (3.0)
**Age**, *M* (*SD*); years	36.31 (13.02)
**Marital status**, *n* (%)	
Single	125 (54.3)
Married/living with partner	85 (37.0)
Divorced/separated	19 (8.3)
Widowed	1 (0.4)
**Living situation**, *n* (%)	
Alone	85 (37.0)
Not alone	145 (63.0)
**Parenthood**, *n* (%)	
Without children	198 (86.1)
With children	32 (13.9)
**Education**, *n* (%)	
< 12 years	83 (36.1)
≥ 12 years	147 (63.9)
**Occupation**, *n* (%)	
Employed	163 (70.8)
Unemployed	67 (29.2)
**Clinician-rated diagnosis**, *n* (%)	
Schizophrenia	26 (11.3)
Bipolar disorder	8 (3.5)
Unipolar depression	91 (39.6)
Anxiety disorder, obsessive-compulsive disorder	61 (26.5)
Personality disorder	13 (5.7)
ADD/ADHD	31 (13.5)

Note. M = mean; *SD* = standard deviation; ADD/ADHD = attention deficit disorder/ attention deficit hyperactivity disorder

To analyse differences in loneliness levels based on participants’ diagnosis, a one-way Analysis of Variance (ANOVA) of all three loneliness scales was conducted.

Further, a multiple linear regression analysis was performed to explore possible associated factors of loneliness. As dependent variable loneliness levels as measured by the UCLA Loneliness Scale were used. The assumptions for regression analysis were analysed. This scale’s scores were used since the UCLA Loneliness Scale is one of the most internationally and widely used questionnaires to measure loneliness. As independent variables the following variables were used: gender, age, living situation, occupation, clinician-rated diagnosis, life satisfaction (SWLS score), need to belong (NTBS score), interpersonal trust (KUSIV3 score), perceived stress (SCI score), and resilience (BRS score). These predictor variables were inserted into the analysis simultaneously. Variables with more than two categories were dichotomized (gender, living alone, living with children, employed, clinician-rated diagnosis). To assess effect sizes, η^2^ and *d* were calculated when appropriate and interpreted as suggested by Cohen [76].

## Results

### Sample characteristics

The study sample consisted of *n* = 230 patients (female: *n* = 132, 57.4%; male: *n* = 91, 39.6%; diverse: *n* = 7, 3.0%). The participants’ age ranged from 18 to 77 years (*M* = 36.31, *SD* = 13.02). The most frequent clinician-rated diagnosis was unipolar depression (*n* = 91, 39.6%), followed by anxiety disorder (*n* = 43, 18.7%) and attention deficit hyperactivity disorder/ attention deficit disorder (*n* = 31, 13.5%). Sociodemographic and clinical characteristics are shown in Table 2.

**Table 2:** Loneliness and social and emotional factors (study sample and comparison with norm samples)

	Study sample (*n* = 230)		Norm sample					
	*n*	*M (SD)*	*n*	*M (SD)*	*t*	*df*	*p*	*d*
Loneliness UCLA-LS UCLA-3-LS De Jong Gierveld LS	230 222 224	47.07 (11.79) 5.84 (2.02) 6.15 (3.55)	492 2182 186	38.90 (10.60) 3.89 (1.34) 4.20 (3.40)	9.31 19.55 5.65	720 2402 408	**< 0.001** **< 0.001** **< 0.001**	0.74 1.38 0.56
Life satisfaction (SWLS)	222	17.77 (6.65)	176	23.50 (6.43)	-8.67	396	**< 0.001**	-0.88
Interpersonal trust (KUSIV3)	221	3.23 (0.86)	1134	3.37 (0.77)	-2.42	1353	**0.016**	-0.18
Perceived stress (SCI)	216	71.06 (22.57)	5220	68.68 (20.90)	1.64	5434	0.102	0.11
Resilience (BRS)	215	2.53 (0.83)	128	3.53 (0.68)	-11.52	341	**< 0.001**	-1.29

Note. *M* = mean; *SD* = standard deviation; *df* = degrees of freedom; *d* = effect size; LS = Loneliness Scale; SWLS = Satisfaction with Life Scale; KUSIV3 = Interpersonal Trust Short Scale; SCI = Stress and Coping Inventory; BRS = Brief Resilience Scale

### Loneliness scores and differences between diagnoses

On the UCLA Loneliness Scale, while only *n* = 42 (18.3%) showed low levels of loneliness, almost half of the respondents reported a moderately high to high degree of loneliness (*n* = 100, 43.5%). On the UCLA 3-item Loneliness Scale, over half of the respondents (*n* = 128, 57.7%) reported to feel lonely. In the third loneliness measure (De Jong Gierveld Loneliness Scale), the participants presented similar numbers: just under a quarter of those surveyed (*n* = 53, 23.7%) reported to not be lonely, whereas *n* = 71 (31.7%) stated severe or even very severe loneliness levels. Results of all three loneliness scales were compared to corresponding norm samples and revealed significantly higher loneliness levels than the general population (all *p* <.001, see Table 1).

To compare loneliness levels between the different diagnoses a one-way ANOVA was performed. Data of the UCLA-LS was normally distributed for each group (Shapiro-Wilk, *p* >.05). Data of the UCLA 3-item LS and the De Jong Gierveld Loneliness Scale was not normally distributed for most diagnosis subgroups (Shapiro-Wilk, *p* <.05), which is why Kruskal-Wallis *H* tests were performed. This analysis showed no significant differences between the six diagnosis subgroups on the UCLA Loneliness Scale, *F*(5, 224) = 2.26, *p* =.049, η^2^ *=* 0.05. To examine potential differences in the other two loneliness scales, Kruskal-Wallis *H* tests were conducted. These analyses showed no significant differences as well for the UCLA 3-item Loneliness Scale, *H*(5) = 5.71, *p* =.335, and for the De Jong Gierveld Loneliness Scale, *H*(5) = 8.40, *p* =.135.

### Social and emotional factors: comparison with norm samples

When compared to norm samples, all except one scale resulted in significant differences. More than half of the participants (*n* = 127, 57.2%) stated that they were slightly to extremely dissatisfied with their life. This outcome equals significantly higher dissatisfaction levels than in a norm sample (*p* <.001, see Table 1). Further, a significantly higher proportion of participants showed low interpersonal trust (*n* = 44, 19.9%) compared to the norm sample (*p* =.016). More than a fifth of the participants reported to feel highly stressed (*n* = 46, 21.3%). Even though these results were higher than in a norm sample, the difference was not statistically significant (*p* =.102). Further, this study’s sample reported significantly lower levels of resilience than a comparative norm sample (*p* <.001). In total, (*n* = 149) 64.8% stated a low resilience, whereas only (*n* = 4) 1.9% reported a high resilience.

### Predictors of loneliness

To examine potential predictors of loneliness, a linear regression analysis was conducted (see Table 3). Linear relationship between variables and homoscedasticity could be assumed. Data were checked for outliers as well as for normal distribution of residuals. The model showed no auto-correlation (Durbin-Watson coefficient = 1.99) and no multicollinearity (*r* ≤.7 in a Pearson correlation matrix as shown in Table 4; Variance Inflation Factor [VIF] ≤ 10). Results indicated that lower life satisfaction, lower interpersonal trust, and lower resilience significantly predicted higher levels of loneliness, *R*^*2*^ = 0.53, *F*(16, 198) = 13.70, *p* <.001. No significant predictive effects were revealed for all other variables entered into the model (all *p* >.05).

**Table 3:** Predictors of loneliness (UCLA Loneliness Scale) in the total sample (*n* = 230)

			95% CI		
	*B*	*SE*	LL	UL	*p*
Male	0.15	3.50	-6.76	7.06	0.966
Female	-0.61	3.40	-7.31	6.09	0.858
Age	0.06	0.05	-0.05	0.16	0.305
Living alone	1.55	1.32	-1.05	4.16	0.241
Living with children	2.30	1.84	-1.34	5.92	0.214
Employed	-1.75	1.47	-4.64	1.15	0.235
Schizophrenia	2.88	3.72	-4.45	10.21	0.440
Unipolar depression	5.65	3.43	-1.12	12.41	0.101
Anxiety disorder, OCD	4.69	3.49	-2.19	11.57	0.181
Personality disorder	3.19	4.27	-5.23	11.60	0.456
ADD/ADHD	3.31	3.71	-4.00	10.63	0.373
Life satisfaction, SWLS	-0.67	0.13	-0.92	-0.42	**< 0.001**
Need to belong, NTBS	1.49	1.23	-0.94	3.93	0.227
Interpersonal trust, KUSIV3	-3.21	0.79	-4.77	-1.65	**< 0.001**
Perceived stress, SCI	0.06	0.03	-0.01	0.13	0.087
Resilience, BRS	-2.05	0.89	-3.81	-0.30	**0.022**
*R*^*2*^ (*R*^*2*^ adjusted)	0.53 (0.49)				
*F*	13.70				
*P*	< 0.001				

Note. B = regression coefficient; *SE* = standard error; CI = confidence interval; *LL* = lower limit; *UL* = upper limit; *R*^*2*^ = coefficient of determination; SWLS = Satisfaction with Life Scale; KUSIV3 = Interpersonal Trust Short Scale; SCI = Stress and Coping Inventory; BRS = Brief Resilience Scale; diagnosis reference categories: bipolar disorder, diverse

**Table 4:** Correlation analysis between the dependent variable (loneliness) and the independent variables

Variables	1	2	3	4	5	6	7	8	9	10	11	12	13	14	15	16	17
1. Loneliness, UCLA-LS	—																
2. Male	− 0.036	—															
3. Female	0.029	− 0.939**	—														
4. Age	− 0.043	0.037	0.021	—													
5. Living alone	0.179**	0.173**	− 0.142*	0.036	—												
6. Living with children	− 0.048	− 0.094	0.118	0.156*	− 0.308**	—											
7. Employed	− 0.150*	− 0.186**	0.164*	− 0.366**	− 0.124	0.064	—										
8. Schizophrenia	− 0.132*	0.132*	− 0.109	0.036	0.096	− 0.104	− 0.194**	—									
9. Unipolar depression	0.156*	− 0.091	0.104	0.077	− 0.048	0.137*	0.010	− 0.289**	—								
10. Anxiety disorder, OCD	− 0.043	0.017	− 0.040	0.039	− 0.031	− 0.014	0.125	− 0.214**	− 0.486**	—							
11. Personality disorder	0.083	− 0.198**	0.173**	− 0.193**	− 0.070	0.010	0.115	− 0.087	− 0.198**	− 0.147*	—						
12. ADD/ADHD	− 0.054	0.149*	− 0.149*	− 0.119	0.041	− 0.048	0.001	− 0.141*	− 0.319**	− 0.237**	− 0.097	—					
13. Life satisfaction, SWLS	− 0.638**	0.042	− 0.022	0.211**	− 0.192**	0.169*	0.129	0.105	− 0.154*	0.112	− 0.089	0.022	—				
14. Need to belong, NTBS	0.145*	− 0.226**	0.188**	− 0.066	0.018	− 0.091	− 0.016	− 0.003	− 0.052	0.001	0.094	− 0.004	− 0.143*	—			
15. Interpersonal trust, KUSIV3	− 0.484**	0.036	− 0.032	0.171*	− 0.094	0.090	0.023	0.082	0.035	− 0.036	− 0.118	− 0.022	0.459**	− 0.065	—		
16. Perceived stress, SCI	0.501**	− 0.063	0.060	− 0.229**	0.101	− 0.017	− 0.010	− 0.150*	0.071	− 0.046	0.110	0.050	− 0.595**	0.189**	− 0.423**	—	
17. Resilience, BRS	− 0.469**	0.236**	− 0.213**	− 0.050	− 0.078	− 0.019	0.103	0.180**	− 0.079	− 0.001	− 0.240**	0.138*	0.458**	− 0.170*	0.357**	− 0.387**	—

Note. *p <.05; **p <.01; LS = Loneliness Scale; SWLS = Satisfaction with Life Scale; NTBS = Need to Belong Scale; KUSIV3 = Interpersonal Trust Short Scale; SCI = Stress and Coping Inventory; BRS = Brief Resilience Scale

## Discussion

The aim of this cross-sectional study was to examine levels of loneliness, social and emotional factors (life satisfaction, interpersonal trust, need to belong, stress, resilience) and potential predictors of loneliness in patients with a psychiatric disorder during the COVID-19 pandemic. To our knowledge, there are hardly any studies on this important global public health topic - even though loneliness as well as mental health had moved very high up on the agenda and public perception in the past three years.

The results showed significantly higher loneliness levels compared to norm samples without psychiatric disorders in all three questionnaires used to investigate loneliness. Thus, the first hypothesis assuming patients with a psychiatric disorder would experience more loneliness than people without psychiatric disorders could be confirmed. Furthermore, the results revealed lower life satisfaction, lower interpersonal trust, and lower resilience in patients with a psychiatric disorder compared to the general population, also confirming the first hypothesis. Further, the six psychiatric diagnosis subgroups show no significant differences in loneliness levels. In addition, lower satisfaction with life, lower interpersonal trust and lower resilience were significant predictors for higher loneliness, partially confirming the second hypothesis.

This study showed that patients with a psychiatric disorder reported significantly higher loneliness levels than people without mental disorders. Even though there are only few studies on the connection between mental disorders and higher loneliness, previous research also confirmed an association between both [34, 43]. This shows that patients with a psychiatric disorder are especially afflicted by loneliness, and the results could build a basis for future research on how to detect or reduce loneliness as early as possible. Further, our findings of lower life satisfaction and lower interpersonal trust in people with a psychiatric disorder were all in line with past literature, which showed that people with mental disorders felt less satisfied with life than the general population [77], and more often reported trust issues [78, 79]. The results on higher stress levels suggest that patients with a psychiatric disorder experience more stress in everyday life compared to people without psychiatric disorders, although these results were not significant. Thus, the results of this study were overall consistent with previous studies [80].

Furthermore, the six psychiatric diagnosis subgroups did not differ in loneliness levels. Often, recent research focussed on one particular psychiatric diagnosis at the time (e.g., patients with depression [40]), whereas this study included a broader spectrum of mental disorders. These results are inconsistent with past research showing that even though patients with psychosis, personality disorders and other mental disorders scored similarly for social isolation, patients with personality disorders showed the highest scores for loneliness, followed by other mental disorders [81]. Interpersonal trust and life satisfaction which were found to be predicting loneliness in the present study were negatively correlated to loneliness in former research [50, 51]. Concerning predictors for loneliness, there are several factors mentioned in other studies, such as resilience [82]. Further, being female, being unemployed and not living with a partner [83] which were found to be risk factors for loneliness in other studies during COVID-19 could not be identified in this study.

When talking about the association between loneliness and mental disorders, the connection can be investigated in both directions: is loneliness the cause or the consequence of mental disorders? Regarding causes of mental disorders, a popular and widely used model is the biopsychosocial model, which aims to explain diseases and disorders by including biological, psychological, and social components [84]. Multiple known reasons why a mental disorder occurs or exacerbates can be comprehended through this model, e.g., genetics, trauma, or drug abuse. Further, social factors can also play an important role in the development of mental disorders. Among other social factors, a lack of social connections and support can result in feelings of isolation and loneliness, which in turn might contribute to the onset or exacerbation of mental disorders. While the biopsychosocial model offers an explanation how loneliness may be a causal factor for mental disorders, it is just as plausible that loneliness may be a consequence of mental disorders. People affected by mental disorders may withdraw from public life, social gatherings, and social connections in general because of their symptoms and the fear of stigma [48]. Findings from a cross-sectional study suggest that stigma around mental disorders may lead to people feeling isolated and hopeless [48]. Since the direction of causality in this study’s results of higher loneliness levels in patients with a psychiatric disorder cannot be specified due to the cross-sectional nature of the study, both explanations seem plausible. Future longitudinal studies may shed light on the multi-faceted associations and causal pathways connecting mental disorders and loneliness.

Regarding loneliness, not only patients’ internal factors for social withdrawal, but also external factors should be taken into consideration. During the COVID-19 pandemic, social isolation was not necessarily self-imposed, but was part of governmental restrictions for confirmed and suspected cases of the virus, and generally in form of social distancing. A French study in patients with a psychiatric disorder revealed that loneliness increased during lockdowns and decreased again when social restrictions were eased [85]. Further, it was found that loneliness was a risk factor for depressive and anxious symptomatology. However, not only people with mental disorders were affected by governmental rules regarding social gatherings. A systematic review with meta-analysis found that there was a small increase in loneliness in the general population during the pandemic compared to before [86]. Further, a rise in depression and anxiety was detected [87]. Another reason why people avoided public life might have been the fear of getting infected. While not being able to meet face-to-face, the importance of social media and the time spent on those platforms to stay in contact increased [88]. However, this development caused more worry, stress and loneliness than amusement since social media provided an extreme amount of information on COVID-19 and the pandemic [89]. In order to avoid loneliness, people increased their social media consumption which in turn lead to higher levels of loneliness, hence describing a vicious cycle [90].

Results of this study found no significant differences in loneliness levels between patients with different psychiatric diagnoses, which may be explained by a variety of reasons. As stated above, research has reported a connection between loneliness and various mental disorders [43]. However, reasons why patients isolate themselves may vary according to the specific diagnosis, e.g., diagnostic criteria for depression include a lack of interest and fatigue, whereas anxiety disorders are associated with an avoidance of certain situations and places. Further, social isolation plays a big part in the clinical picture of schizophrenia [91]. Therefore, the symptomatology associated with specific psychiatric disorders may lead to social withdrawal, therefore potentially leading to loneliness. The assumption that all psychiatric disorders can lead to loneliness could explain why no significant differences in loneliness levels between different psychiatric diagnoses were found.

In the present study, satisfaction with life, interpersonal trust, and resilience were found to be predictors of loneliness. The personality theory concept of state and trait may explain why. A state describes a person’s characteristic pattern in behaviour and thinking in a specific situation, and is rather short-lived [92]. In contrast, a trait is an enduring (sometimes lifelong) characteristic pattern in behaviour and thinking that applies in similar situations over time [92]. Interpersonal trust and resilience can be considered as traits, while loneliness would more likely fit the description of a state. Thus, our findings might suggest that loneliness results from a decreased interpersonal trust, since people socialise less because it might be harder for them to trust others. Further, it could mean that because of a lower resilience and therefore a reduced ability to withstand or recover from distress, people may feel lonelier. Establishing causality in these findings is limited because of the cross-sectional study design.

One of this study’s strengths was the large sample size and the sample’s clinical diversity, consisting of inpatients, outpatients, and day clinic patients with various mental disorders. The fact that standardised measures were used allows the option to extend or replicate the study, or more easily compare it to future research. Three different questionnaires were included in this study to thoroughly assess the construct of loneliness. The limitations of this study include the possibility of self-selection bias and the inability to determine causality because of the cross-sectional study design. Both should be considered when interpreting the results. Furthermore, the comparability of diagnosis groups is limited because of varying sample sizes for the respective diagnoses. Further, potential effects from taking medication or receiving psychotherapy on patients’ emotional experience of loneliness cannot be ruled out and should be explored in future studies. Another limitation that should be considered is that some of the norm samples that were used to compare the participants’ results differed from the psychiatric sample in sample characteristics such as age or gender. That means that our results should be applied to a rather specific setting. Nonetheless this study should be considered very valuable since it sheds light on an under-represented group of people regarding research.

Moreover, questionnaires examining trust should be conducted more often in everyday clinical practice to identify people with lower interpersonal trust, even before they report loneliness, since our study showed that those with lower trust were more at risk to be lonely. A good relationship between patient and health staff should form the base of further examining where these trust issues stem from in therapy. In addition, interpersonal trust and its origins in patients with a psychiatric disorder might prove an important area for future research.

## Conclusion

In summary, loneliness is a worldwide issue which attracted even more attention during the COVID-19 pandemic. Research on loneliness in patients with a psychiatric disorder is to date rather sparse. The study provides insight into the concept of loneliness and most importantly throws light on this group of patients. By investigating loneliness in patients with a psychiatric disorder, this study suggests that people with mental disorders experience significantly more loneliness than the general population. Further, low satisfaction with life, low resilience and low interpersonal trust appear to be associated factors of loneliness. To transfer the study’s findings into clinical practice, ways to prevent or lower loneliness in patients with a psychiatric disorder should be investigated. Specifically, low-threshold services that invite people with mental disorders to come together and connect are needed. A meta-analysis found positive effects for shared interest groups, videoconferencing interventions, and befriending services, but still concluded that further research on such interventions needs to be done [93]. Studies on preventing loneliness particularly in people with mental disorders showed promising results for digital solutions [94] (smartphone application) and supported housing, where tenants lived in independent units in apartment buildings including common areas and support staff [95]. In Germany, some of these interventions were already in place, e.g. low-threshold peer-counselling and self-help projects and could be upgraded to meet patients’ diverse needs for company e.g., in the form of online programs. Further, longitudinal studies may provide meaningful insights into associations and causal effects of loneliness in patients with a psychiatric disorder.

## Data Availability

All relevant data files are available from the OSF database (DOI 10.17605/OSF.IO/RWC94).
